# At the Crossroads of Synapses and Signaling: The Role of LRRK2 in Neuronal Communication

**DOI:** 10.1007/s10571-026-01793-9

**Published:** 2026-07-29

**Authors:** Angela Di Iacovo, Chiara D’Agostino, Giulia Casoli, Tiziana Romanazzi, Elena Bossi, Cristina Roseti

**Affiliations:** 1 Department of Biotechnology and Life Sciences, University of Insubria, Via Jean Henry Dunant, 3, 21100 Varese, Italy; 2 Centre for Neuroscience, University of Insubria, 21100 Varese, Italy; 3 PhD School of Experimental and Translational Medicine, University of Insubria, 21100 Varese, Italy

**Keywords:** Glutamate receptor, GABA receptor, Excitatory/inhibitory balance, Neurotransmitters transporters, Parkinson’s disease

## Abstract

Precise control of membrane protein localization and turnover is essential for synaptic function and circuit stability. Disruption of these processes alters the surface expression of receptors and transporters, ultimately impacting the excitatory/inhibitory (E/I) balance. Leucine-rich repeat kinase 2 (LRRK2) is emerging as a key regulator of these membrane-associated processes, acting at the interface between intracellular trafficking and synaptic signaling. LRRK2 integrates kinase activity with scaffolding functions to coordinate Rab-dependent vesicular trafficking, endosomal recycling, cytoskeletal dynamics, and endolysosomal pathways. Through these mechanisms, it controls the surface expression and distribution of synaptic receptors and neurotransmitter transporters in both neuronal and glial compartments. Pathogenic LRRK2 variants associated with Parkinson’s disease (PD), converge on kinase hyperactivation and lead to sustained alterations in trafficking networks, resulting in selective mislocalization of membrane proteins. In this review, we examine how LRRK2-dependent trafficking mechanisms regulate key components of synaptic transmission, including glutamatergic and GABAergic receptors, as well as astrocytic transporters. We highlight how disruption of these processes affects neurotransmitter clearance, receptor activation, and ultimately E/I balance. Notably, accumulating evidence indicates that LRRK2 functions as an upstream coordinator of membrane organization at the crossroads of synaptic signaling and neurotransmitter homeostasis. Understanding how LRRK2 regulates trafficking pathways across cellular compartments provides mechanistic insight into circuit vulnerability in PD and highlights membrane-targeted strategies as potential approaches for restoring synaptic stability.

**Graphical Abstract:**

LRRK2 in Synaptic Communication.

LRRK2 acts as a central regulator of membrane trafficking in neurons and astrocytes. Through control of vesicular pathways, cytoskeletal dynamics, and endolysosomal function, LRRK2 modulates synaptic receptors and neurotransmitter transporters, contributing to excitatory/inhibitory balance. Pathogenic variants disrupt these processes, promoting synaptic dysfunction and circuit instability

**Figure:**
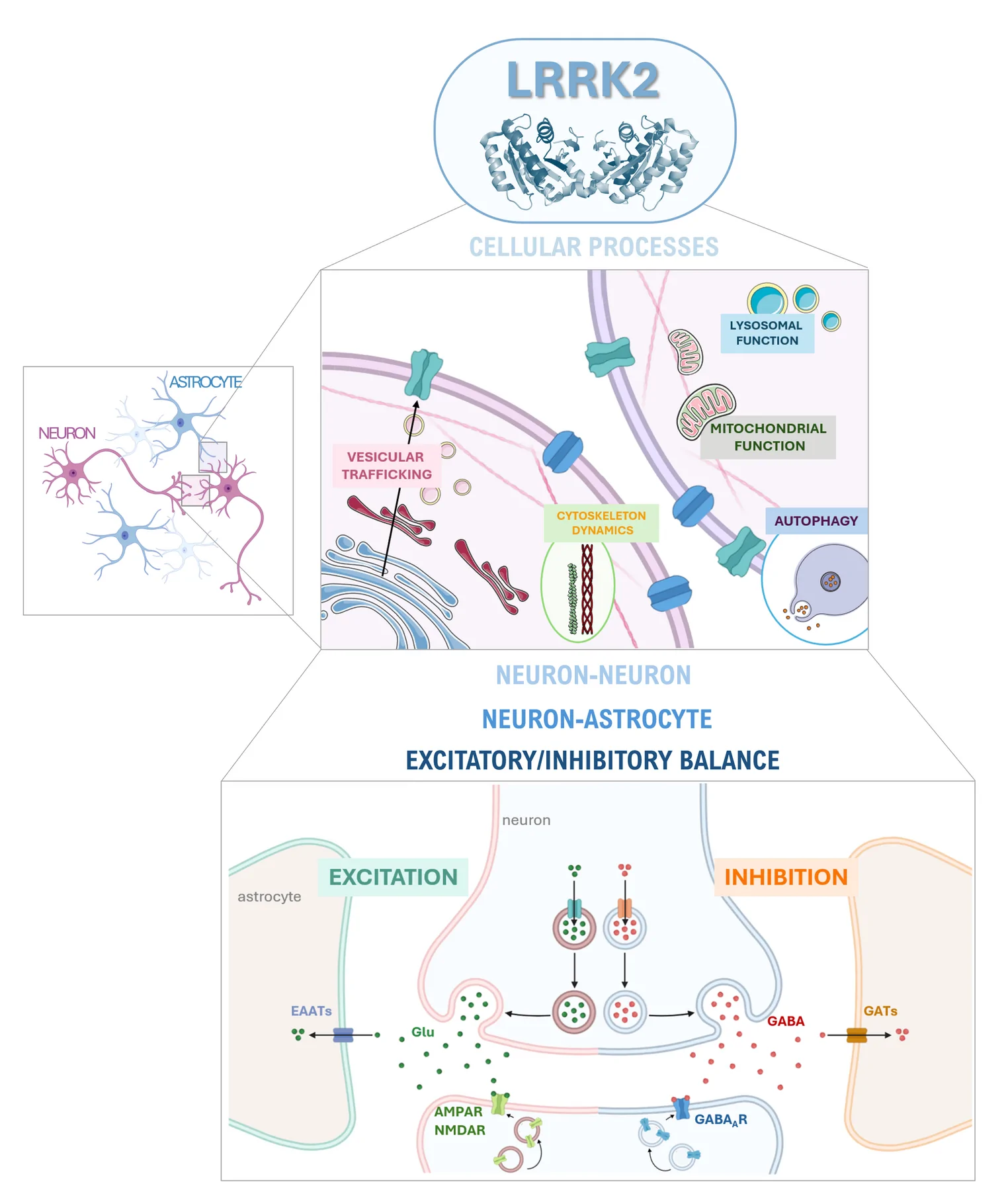

## LRRK2

### Structural Organization and Pathogenic Variants of LRRK2

Precise control of membrane protein localization is essential for synaptic communication. The density, retention, and turnover of receptors and transporters at the cell surface directly determine neuronal excitability and circuit stability. Within this framework, LRRK2 has emerged as a key regulator linking intracellular trafficking to the surface expression of proteins that govern excitatory and inhibitory balance.

LRRK2 is widely expressed throughout the central nervous system, in both neurons and glial cells such as astrocytes and microglia, positioning it to influence both synaptic and perisynaptic membrane systems (Miklossy et al. 2006). Despite uncertainties regarding its precise role, LRRK2 has been implicated in neurite outgrowth, vesicle trafficking, cytoskeletal organization, autophagy, and immune regulation (Kluss et al. 2019).

Since its identification as a major genetic determinant of Parkinson’s disease (PD), LRRK2 has been extensively studied because of its dual enzymatic activity, combining serine/threonine kinase function with GTPase activity, as well as for its role as a scaffold within multiprotein signaling complexes.

Structurally, LRRK2 is a multidomain protein composed of N-terminal ankyrin and leucine-rich repeats, followed by the ROC–COR GTPase tandem, the kinase domain, and a C-terminal WD40 domain. This organization supports both catalytic regulation and assembly into higher-order signaling complexes. Importantly, the ROC–COR module and the kinase domain are functionally interconnected: kinase activity is enhanced in the GTP-bound state of the ROC domain, indicating a GTP-dependent mechanism of activation (Li et al. 2014). In addition, dimerization, primarily mediated by ROC–COR interactions, stabilizes the active conformation and is required for efficient kinase signaling (Civiero et al. 2012).

LRRK2 variants are implicated in both familial and sporadic PD, with pathogenic substitutions clustering predominantly within the catalytic core. The most common mutation, G2019S, located in the kinase domain, consistently increases kinase activity approximately two- to threefold (West et al. 2005; Jaleel et al. 2007; Greggio et al. 2006). Nearby variants such as I2020T also enhance kinase output, although with more variable effects.

In contrast, mutations in the ROC and COR domains (including R1441C/G/H, N1437H, and Y1699C) primarily impair GTPase activity. These alterations reduce GTP hydrolysis and prolong the GTP-bound state of LRRK2, indirectly promoting kinase activation and disrupting intramolecular regulation (Lewis et al. 2007; Li et al. 2007; Liao et al. 2014; Huang et al. 2019). Despite these mechanistic differences, pathogenic variants converge on a common outcome: kinase hyperactivation and aberrant phosphorylation of downstream substrates.

Importantly, these alterations extend to synaptic physiology, where mutation-specific effects have been observed across multiple experimental models (Beccano-Kelly et al. 2014; Matikainen-Ankney et al. 2016; Volta et al. 2017).

Taken together, LRRK2 mutations should not be viewed merely as genetic risk factors, but as drivers of widespread perturbations in membrane-associated signaling networks. By disrupting the coordination between GTPase activity, kinase signaling, and Rab-mediated trafficking, these variants ultimately alter the localization and stability of membrane proteins, that are particularly critical at synapses.

### LRRK2 as a Regulator of Membrane Protein Dynamics and Synaptic Balance

LRRK2 integrates kinase activity with scaffolding functions to coordinate intracellular trafficking pathways that govern membrane protein distribution in neurons and glial cells. Beyond its enzymatic role, LRRK2 acts as an organizer of trafficking networks, regulating the localization, stability, and turnover of receptors and transporters that are essential for synaptic signaling(Shin et al. 2008; Bonet-Ponce and Cookson 2022; Pfeffer 2013).

A major advance in the field has been the identification of Rab GTPases as direct LRRK2 targets, linking LRRK2 activity to endosomal trafficking, vesicle dynamics, and membrane recycling pathways. Through phosphorylation of Rab GTPases, LRRK2 controls key steps of vesicular trafficking, including endocytosis, recycling, and surface retention (Steger et al. 2016; Pfeffer 2013; Iovino et al. 2022). Rather than acting on isolated substrates, this regulatory activity operates at the level of membrane systems, shaping the distribution of proteins across cellular compartments. In LRRK2 mutant models, persistent Rab hyperphosphorylation supports the idea that pathogenic activation leads to sustained rewiring of trafficking pathways rather than transient perturbations (Steger et al. 2016).

The precise surface expression of receptors, transporters, adhesion molecules, and scaffolding proteins is essential for maintaining neuronal excitability and circuit stability. In this context, excitatory/inhibitory (E/I) balance represents a sensitive functional readout of membrane homeostasis.

Consistent with this view, electrophysiological studies in LRRK2 knock-in models reveal early circuit-level alterations that precede overt neurodegeneration (Beccano-Kelly et al. 2014; Matikainen-Ankney et al. 2016; Volta et al. 2017). These findings support the idea that defects in membrane trafficking and protein distribution represent primary drivers of synaptic imbalance.

Together, these observations position LRRK2 as a central regulator of trafficking systems that link intracellular transport dynamics to synaptic function and circuit stability.

### LRRK2 as a Regulator of Membrane Trafficking and Surface Protein Stability

LRRK2 exerts its effects through direct control of intracellular trafficking pathways that determine membrane protein sorting and fate. Phosphorylation of Rab8A, Rab10, Rab12, and related isoforms alters their interactions with regulatory proteins, thereby influencing membrane association and vesicular targeting (Waschbüsch et al. 2014; Liu et al. 2018; Pfeffer 2013). Pathogenic variants such as G2019S enhance this activity, leading to increased Rab phosphorylation and altered endosomal dynamics (Steger et al. 2016; Eguchi et al. 2018).

Experimental models expressing hyperactive LRRK2 display enlarged endosomes, delayed cargo recycling, and altered lysosomal positioning, consistent with a global disruption of trafficking pathways (Kuwahara and Iwatsubo 2020; Bonet-Ponce and Cookson 2022).

In addition to Rab-mediated mechanisms, LRRK2 interacts with other components of the trafficking machinery. It associates with dynamin isoforms on early endosomal membranes, modulating scission events required for clathrin-mediated endocytosis and vesicle recycling (Matta et al. 2012). Similarly, LRRK2 influences Rab5-dependent early endosomal dynamics, supporting a role in the initial stages of membrane internalization (Shin et al. 2008). Its regulatory scope further extends to the trans-Golgi network through Rab7L1-dependent recruitment, affecting both retrograde and anterograde trafficking between intracellular compartments and the plasma membrane (MacLeod et al. 2013; Purlyte et al. 2019; Liu et al. 2018).

A further layer of control involves autophagy. LRRK2 phosphorylates membrane-remodeling proteins such as Endophilin A, promoting the generation of curved membranes required for autophagosome formation, particularly at presynaptic sites (Soukup et al. 2016). Notably, LRRK2 activity is spatially restricted: only membrane-associated, GTP-bound Rab proteins are efficiently phosphorylated, indicating a strong compartment-specific component of its function (Steger et al. 2016).

Importantly, Rab phosphorylation is dynamically counterbalanced by phosphatase-mediated dephosphorylation, which restores Rab localization and activity. This reversible cycle provides temporal control over membrane trafficking events and prevents persistent pathway activation. Recent studies have highlighted the importance of this regulatory equilibrium, suggesting that pathological consequences of LRRK2 hyperactivity may arise not only from excessive Rab phosphorylation but also from impaired dephosphorylation mechanisms (Berndsen et al. 2019; Dou et al. 2024).

LRRK2 also interfaces with the cytoskeleton. Active LRRK2 associates with microtubules in a kinase-dependent manner and can form filamentous structures that may influence motor protein access and vesicle transport directionality (Deniston et al. 2020). This structural coupling provides a mechanistic basis for coordinated trafficking across neuronal and glial compartments, and alterations translate into defects in surface protein stability. For example, in primary astrocytes, the G2019S variant promotes accumulation of the glutamate transporter EAAT2 in early endosomes, reducing its surface expression in a kinase-dependent manner reversible by pharmacological inhibition (Iovino et al. 2022).

In addition to its well-established functions in endocytic and recycling pathways, accumulating evidence suggests that LRRK2 also regulates SNARE-dependent secretory trafficking. LRRK2 has been shown to interact with components of the Golgi-associated retrograde protein (GARP) complex, thereby influencing retrograde transport to the trans-Golgi network and membrane fusion events involving the v-SNARE VAMP4 (Beilina et al. 2020). Furthermore, genetic studies have identified VAMP4 as a potential modifier of LRRK2-associated Parkinson’s disease, supporting a functional connection between secretory trafficking pathways and disease progression (Brown et al. 2021). Beyond the Golgi apparatus, LRRK2 plays an important role in endolysosomal membrane dynamics, where it regulates Rab-dependent trafficking events that influence lysosomal positioning, membrane remodeling, and vesicle fusion. Given the emerging recognition of lysosomes as secretory organelles, these mechanisms may also contribute to lysosomal exocytosis, extracellular vesicle release, and unconventional secretion pathways, processes increasingly implicated in intercellular communication and neurodegeneration (Erb and Moore 2020; Bonet-Ponce and Cookson 2019). These observations suggest that LRRK2 coordinates both degradative and secretory trafficking networks, extending its influence beyond membrane protein turnover to the regulation of extracellular signaling.

Collectively, these findings position LRRK2 as an upstream coordinator of membrane protein stability. By regulating vesicular routing, recycling efficiency, degradation, and spatial organization of membranes, LRRK2 ensures proper synaptic signaling and neurotransmitter clearance. Pathogenic variants disrupt this coordination, leading to selective surface protein dysregulation and increased circuit vulnerability.

## LRRK2 in Glial Cells and Neuroinflammatory Signaling

Although LRRK2 has been extensively studied in neurons, increasing evidence indicates that its expression in glial cells plays a significant role in the regulation of brain homeostasis and inflammatory signaling. LRRK2 is highly expressed in immune cells and in glial populations such as microglia and astrocytes, where it participates in processes related to vesicle trafficking, cytoskeletal remodeling, and inflammatory responses (Cookson 2010; Moehle et al. 2012; Russo et al. 2014).

Microglia are the primary immune effector cells of the CNS, and LRRK2 expression in these cells is dynamically modulated by inflammatory stimuli. Exposure to lipopolysaccharide (LPS) or interferon-γ induces robust upregulation of LRRK2 expression in microglia and macrophages, suggesting a role for the kinase in innate immune signaling pathways (Moehle et al. 2012; Gardet et al. 2010). In these contexts, LRRK2 participates in the regulation of vesicular trafficking events that control cytokine secretion and phagocytic activity. Experimental studies have shown that LRRK2 kinase activity modulates microglial motility, chemotaxis, and phagosome maturation, linking its trafficking functions to immune surveillance mechanisms (Ma et al. 2016; Choi et al. 2015).

Pathogenic LRRK2 variants can alter these processes. Microglia expressing the G2019S mutation exhibit enhanced inflammatory responses, including increased production of pro-inflammatory cytokines and altered phagocytic dynamics (Moehle et al. 2012; Russo et al. 2014). These effects appear to be mediated, at least in part, through dysregulated Rab phosphorylation and consequent alterations in vesicle trafficking pathways involved in immune signaling. Rab8A and Rab10, which are well-established substrates of LRRK2, participate in endosomal and recycling pathways that regulate receptor trafficking and membrane delivery during immune activation (Steger et al. 2016); Pfeffer 2013).

Astrocytes also represent a critical cellular context in which LRRK2 activity influences membrane protein regulation. Beyond their well-known role in neurotransmitter clearance, astrocytes actively contribute to synapse formation, metabolic support, and neuroinflammatory signaling, positioning them as key regulators of synaptic homeostasis.

Emerging evidence indicates that LRRK2 activity in astrocytes directly affects vesicle trafficking pathways that control membrane protein localization, recycling, and degradation. In particular, LRRK2-dependent regulation of Rab GTPases has been linked to alterations in endosomal dynamics that affect the surface stability of astrocytic membrane proteins involved in neurotransmitter clearance. Consistent with this, pathogenic LRRK2 variants have been shown to disrupt the trafficking and membrane retention of key astrocytic transporter, ultimately impairing glutamate homeostasis in a kinase-dependent manner (Iovino et al. 2022). Through this mechanism, astrocytic LRRK2 activity can indirectly influence neuronal excitability and contribute to excitatory/inhibitory balance by regulating extracellular neurotransmitter dynamics. In addition, LRRK2 activity in astrocytes has been associated with modulation of reactive states and inflammatory signaling (He et al. 2023), suggesting a broader role in shaping the extracellular environment and neuron–glia communication.

Notably, astrocyte and microglial communication relies heavily on secretory pathways, including cytokine release, lysosomal exocytosis, and extracellular vesicle-mediated signaling. Beyond its established roles in endocytic and degradative pathways, increasing evidence indicates that LRRK2 also regulates secretory trafficking mechanisms. LRRK2 interacts with post-Golgi v-SNARE-dependent pathways involving VAMP4 and VAMP7 and modulates cargo secretion through endosomal and lysosomal compartments (Filippini et al. 2023). Furthermore, recent studies have shown that lysosomal stress promotes LRRK2 recruitment and activation at lysosomal membranes, triggering Rab-dependent trafficking responses that facilitate extracellular secretion of lysosomal contents and pathogenic cargoes, including α-synuclein (Eguchi et al. 2018; Abe et al. 2024). These observations support an emerging role for LRRK2 as a regulator of secretory trafficking and intercellular communication, extending its influence beyond membrane protein turnover to mechanisms that may contribute to neuron–glia communication in both physiological and pathological conditions.

These findings highlight that LRRK2 operates across neuronal and glial compartments, shaping synaptic communication through coordinated regulation of receptor localization and neurotransmitter clearance mechanisms.

## Excitatory/Inhibitory Imbalance as a Disease Mechanism

The balance between excitatory/inhibitory (E/I) signaling is essential for stable neuronal activity and efficient information processing. This balance is maintained through the precise regulation of glutamatergic and GABAergic transmission mediated by the coordinated activity of synaptic receptors and neurotransmitter transporters. Disruption of E/I homeostasis is increasingly recognized as an early feature of both neurodevelopmental and neurodegenerative disorders, including PD (Ghatak et al. 2021). Even subtle alterations in receptor density, synaptic strength, or neurotransmitter clearance can destabilize circuit function and contribute to neurological disorders (Sears and Hewett 2021).

Rather than being solely a consequence of neuronal loss, E/I imbalance may arise from primary alterations in the molecular mechanisms that control synaptic signaling. In this context, proteins regulating membrane trafficking are critical, as they determine the availability and distribution of receptors and transporters that shape synaptic input and output.

Within this framework, LRRK2 emerges as an upstream modulator capable of influencing circuit stability by regulating the trafficking-dependent control of synaptic components, including both receptors and neurotransmitter transporters.

## LRRK2 and Synaptic Receptors: Excitatory and Emerging Inhibitory Regulation

Synaptic receptors are central determinants of neuronal excitability and network function. LRRK2 has emerged as a critical regulator of receptor trafficking, surface stability, and synaptic efficacy.

The role of LRRK2 in excitatory synaptic transmission has been extensively investigated, and alterations in glutamatergic signaling represent one of the most consistently reported phenotypes across LRRK2 models. At postsynaptic sites, LRRK2 modulates the trafficking and surface expression of AMPA-type glutamate receptors (AMPARs), influencing synaptic strength and plasticity.

Parisiadou et al. reported that striatal slices from LRRK2 knock-out (KO) pups exhibited reduced frequency of spontaneous excitatory postsynaptic currents (sEPSCs), accompanied by increased PKA-mediated phosphorylation of the AMPA GluA1 subunit (Parisiadou et al. 2014). The G2019S variant has been associated with altered excitatory synaptic transmission, changes in AMPAR trafficking, and impaired synaptic plasticity. In LRRK2 G2019S knock-in (KI) mice, Beccano-Kelly et al. reported an approximately 35% increase in miniature excitatory postsynaptic currents (mEPSCs) frequency in cortical neurons relative to control mice (Beccano-Kelly et al. 2014). Volta et al. further confirmed elevated sEPSC frequency in striatal slices from G2019S KI mice at 1–3 months of age, with normalization in adult mice (> 12 months), parallel with dynamic changes in dopamine neurotransmission (Volta et al. 2017). In vitro studies in cortical neurons expressing G2019S or R1441C LRRK2 show increased AMPA- and NMDA-mediated inward currents, along with elevated VGLUT1 and PSD-95 puncta density, indicative of aberrant glutamatergic synapse number and function (Beccano-Kelly et al. 2014); Plowey et al. 2014). Recently, Gupta and colleagues demonstrated that the LRRK2-G2019S mutation disrupts not only AMPAR trafficking but also receptor mobility and subunit composition in the striatum. These findings extend current knowledge of LRRK2-dependent modulation of glutamatergic transmission and revealing a previously underappreciated role of LRRK2 in striatal excitatory synaptic function (Gupta et al. 2024).

The hyperactivity of AMPA- and NMDA-receptors is also validated in *Xenopus laevis* oocytes microtransplanted with striatal membranes from LRRK2 G2019S KI-mice (Di Iacovo et al. 2025a, ). Importantly, the neurodegenerative effects of mutant LRRK2 on dendrites were mitigated by memantine, an NMDA receptor antagonist, highlighting the functional relevance of NMDA receptor dysregulation (Sweet et al. 2015). Mechanistically, these effects appear to stem from post-translational modulation of receptor trafficking and surface retention rather than transcriptional changes, suggesting that LRRK2 establishes baseline excitatory tone through control of receptor dynamics at synapses (Volta et al. 2017; Beccano-Kelly et al. 2014). While excitatory receptor dysregulation has been extensively studied, inhibitory synapses remain comparatively underexplored and may play a central role in LRRK2-driven E/I imbalance. Recent evidence indicates that LRRK2 G2019S negatively modulates GABA_A_ receptor exposure on striatal cell membranes (Di Iacovo et al. 2025a). These findings highlight a novel layer by which synaptic inhibition could be compromised by LRRK2 (Di Iacovo et al. 2025a). Although data on inhibitory synapses are still emerging, these findings open new perspectives for understanding how LRRK2 mutations shift E/I balance through coordinated modulation of both excitatory and inhibitory synapses, extending the functional impact beyond glutamatergic circuits. Together with the recent extension of LRRK2-dependent glutamatergic modulation to the striatum (Gupta et al. 2024), these observations further support this circuit as a key hub of LRRK2-mediated synaptic regulation, potentially integrating both excitatory and inhibitory mechanisms.

## From Synaptic Receptor to Neurotransmitter Transporters: The Role of LRRK2

Synaptic receptor function is tightly regulated by extracellular neurotransmitter availability, which is determined not only by presynaptic release but also by uptake mechanisms mediated by transporters expressed in neurons and glial cells. Impaired reuptake of glutamate or GABA prolongs neurotransmitter residence in the extracellular space, facilitating spillover and altering receptor activation profiles, ultimately perturbing the balance between excitatory and inhibitory signaling. In this context, dysfunction of neurotransmitter transporters is increasingly recognized as a complementary mechanism that can exacerbate synaptic imbalance.

These considerations highlight astrocytic and neuronal transporters as key contributors to the regulation of E/I balance. In line with this view, emerging evidence indicates that LRRK2 may influence neuronal excitability not only through the regulation of receptor trafficking, but also by modulating extracellular neurotransmitter dynamics. In particular, LRRK2 has been implicated in the control of astrocytic glutamate clearance, with potential downstream consequences for E/I balance. In addition to its effects on glutamatergic and inhibitory neurotransmission, LRRK2 has also emerged as an important regulator of dopaminergic signaling.

Several studies have implicated LRRK2 in the regulation of dopaminergic neurotransmission and DAT function, suggesting that alterations in DAT trafficking and membrane dynamics may contribute to abnormal dopamine homeostasis in Parkinson’s disease (Longo et al. 2017; Bu et al. 2021; Brzozowski et al. 2021). However, evidence from heterologous expression systems suggests that this effect may depend on the cellular context, indicating that additional trafficking regulators could be required for LRRK2-dependent modulation of DAT function (Di Iacovo et al. 2025b). Thus, several findings links LRRK2 to DAT-dependent dopaminergic dysfunction, although the extent to which LRRK2 directly regulates DAT trafficking and recycling remains to be fully established. These observations extend the impact of LRRK2-dependent trafficking beyond excitatory/inhibitory balance and further support its role as a broader regulator of synaptic homeostasis in Parkinson’s disease. In this context, an additional layer of complexity is provided by α-synuclein, another key Parkinson’s disease-associated protein that regulates synaptic vesicle dynamics and membrane trafficking.

Alpha-synuclein has been implicated in the regulation of DAT trafficking and surface expression, suggesting that both α-synuclein and LRRK2 converge on common pathways controlling membrane protein turnover and synaptic homeostasis. The interplay between these proteins may therefore represent an important determinant of neurotransmitter imbalance and circuit dysfunction in Parkinson’s disease.

### Astrocytic Glutamate Clearance and EAAT2 Dysfunction in LRRK2-Related PD

Glutamate accumulation into the synaptic cleft over physiological range is toxic and induces excessive calcium influx, leading to apoptotic cell death. The so-called excitotoxicity phenomenon is a well-established hallmark of several neurodegenerative diseases (Lewerenz and Maher 2015). Accordingly, the regulation of extracellular glutamate levels is crucial to ensure the physiological excitatory signalling. Efficient glutamate clearance is primarily mediated by EAAT2, the most abundant glutamate transporter in the forebrain, responsible for ~ 80–90% of extracellular glutamate uptake. EAAT2 ensures rapid restoration of basal glutamate concentrations (< 25 nM) following synaptic release (~ 1 mM), preventing excitotoxic activation of NMDA receptors, particularly at extrasynaptic sites (Otis and Jahr 1998; Hardingham and Bading 2010). 

Multiple experimental PD models consistently report downregulation of the astrocytic glutamate transporter Glt-1 (human EAAT2), accompanied by extracellular glutamate accumulation and neuroinflammatory activation. Such alterations have been observed in toxin-based models including 6-hydroxydopamine (6-OHDA) and 1-methyl-4-phenyl-1,2,3,6-tetrahydropyridine (MPTP), as well as in genetic models including A53T α-synuclein mice, where Glt-1 and GLAST (human EAAT1) are down-regulated (Gu et al. 2010; Chung et al. 2009) . While these studies establish EAAT2 dysfunction as a recurrent feature of PD, they do not identify the upstream mechanisms governing its membrane instability. In this context, LRRK2 provides a mechanistic entry point. Under physiological conditions, LRRK2 regulates EAAT2 activity promoting its membrane localization at cell surface (Di Iacovo et al. 2025b). Iovino and colleagues demonstrated that the pathogenic G2019S variant promotes transporter accumulation in Rab4-positive early endosomes, reducing Glt-1 surface availability in astrocytes. This effect is kinase-dependent and reversible by pharmacological inhibition, directly linking LRRK2 hyperactivity to impaired glutamate clearance (Iovino et al. 2022). Thus, beyond the general association between glutamate dyshomeostasis and PD, LRRK2 emerges as a specific regulator of EAAT2 membrane stability. Importantly, this modulation appears highly selective for EAAT2: other transporters examined in comparable systems, including EAAT1, are unaffected (Di Iacovo et al. 2025b). Structural divergence within the EAAT2 C-terminal domain, enriched in regulatory motifs, may contribute to its isoform-specific sensitivity, although the precise molecular interface with LRRK2-dependent trafficking pathways remains to be clarified.

Together, these findings support a revised interpretation of glutamate dyshomeostasis in PD. Rather than solely representing a downstream consequence of neurodegeneration, impaired EAAT2 membrane stability may also reflect an upstream trafficking defect associated with pathogenic LRRK2 activity. Reduced EAAT2 localization at perisynaptic astrocytic processes is expected to impair spatial buffering of glutamate, thereby promoting synaptic spillover and altering synaptic integration. These alterations may, in turn, contribute to maladaptive synaptic responses and increased circuit vulnerability in LRRK2-associated PD.

### Coupling Between Glutamate and GABA Transporters

A recent clinical study reported lower GABA levels in the upper brainstem of PD patients, suggesting that GABAergic deficit may contribute to the pathology in PD (Song et al. 2021). The mechanisms underlying the GABA deficit remain unclear, and the available evidence is still limited. GABA transporters (GATs), primarily GAT-1 and GAT-3, regulate extracellular GABA levels and shape inhibitory tone through Na⁺- and Cl⁻-dependent uptake mechanisms. Notably, astrocytic GAT-3 operates in close functional association with the glutamate transporter EAAT2, as both transporters share dependence on the intracellular Na⁺ gradient. Glutamate uptake via EAAT2 leads to intracellular Na⁺ accumulation, which can influence other Na⁺-coupled transporters expressed in astrocytes. In this context, increased intracellular Na⁺ and GABA levels may promote reversal of GAT-3 transport direction, resulting in GABA efflux and activation of extrasynaptic GABA receptors, thereby contributing to tonic inhibition (Héja et al. 2009, 2012; Flanagan et al. 2021). This hetero-exchange mechanism represents a form of astrocyte-mediated feedback coupling excitatory clearance to inhibitory modulation, potentially acting as a compensatory brake during excessive synaptic excitation.

Alterations in GAT function have been implicated in neurodegenerative disorders, including Huntington’s disease and depression associated with PD, suggesting that disrupted inhibitory transporter function may amplify excitatory-dominant circuit phenotypes (Wójtowicz et al. 2013). However, experimental evidence in *Xenopus laevis* oocyte systems shows that LRRK2 WT or G2019S do not significantly alter GAT-1 activity, suggesting that immediate, post-translational modulation of these inhibitory transporters is not evident (Di Iacovo et al. 2025b). Whether LRRK2 influences GAT-3 remains unknown.

These findings indicate that, in this heterologous system, LRRK2 does not directly modulate the tested inhibitory transporters, contrasting with its pronounced effect on EAAT2. Nevertheless, given the tight functional coupling between EAAT2 and GAT-3 in astrocytes, LRRK2-dependent modulation of glutamate transport could indirectly impact GABA dynamics.

### Glycine Transporters: Emerging but Unexplored Layer of Inhibitory Control

GlyT1 and GlyT2 control extracellular glycine levels, contributing to inhibitory neurotransmission and NMDA receptor co-agonism. In oocytes system, neither LRRK2 WT nor G2019S altered GlyT1 or GlyT2 activity (Di Iacovo et al. 2025b). Although no direct effect is observed in Xenopus oocytes system, this does not preclude LRRK2-dependent modulation in more complex cellular contexts. Given glycine’s role in NMDA receptor gating and LRRK2’s impact on NMDA receptor, it is plausible that GlyT function could be indirectly influenced under physiological or pathological conditions.

## Therapeutic Implications: Targeting LRRK2-Dependent Trafficking Pathways

The central role of LRRK2 in vesicle trafficking and membrane protein regulation has stimulated considerable interest in therapeutic strategies aimed at modulating its kinase activity. Among the approaches under investigation, small-molecule inhibitors targeting the LRRK2 kinase domain represent a promising class of therapeutics currently in clinical development. (West 2017); Tolosa et al. 2020). Pharmacological inhibition of LRRK2 kinase activity effectively reduces phosphorylation of Rab substrates such as Rab8A and Rab10, thereby restoring trafficking dynamics in cellular and animal models of PD (Steger et al. 2016; Deniston et al. 2020). Several LRRK2 kinase inhibitors have demonstrated the ability to normalize Rab phosphorylation levels and rescue trafficking defects associated with pathogenic LRRK2 variants, supporting the therapeutic potential of targeting this signaling axis (Steger et al. 2016; Deng et al. 2008).

In addition to direct kinase inhibition, modulation of downstream trafficking pathways represents an emerging therapeutic concept. Because LRRK2 activity affects multiple membrane systems, including endosomal recycling, lysosomal degradation, and autophagy, interventions that restore these pathways may help stabilize synaptic protein localization and prevent early circuit dysfunction. For example, pharmacological modulation of lysosomal function or autophagy has been shown to ameliorate trafficking defects and improve neuronal viability in models of LRRK2-associated neurodegeneration (Manzoni and Lewis 2017; Madureira et al. 2020; Pang et al. 2022). Beyond degradative pathways, the involvement of LRRK2 in secretory trafficking pathways further suggests that these processes may represent additional therapeutic targets for restoring synaptic and intercellular homeostasis.

Interestingly, recent evidence demonstrating LRRK2-dependent modulation of GABAergic signaling has been obtained in heterologous expression system, indicating that these alterations may occur independently of disease progression and neurodegeneration (Di Iacovo et al. 2025a, b). This suggests that LRRK2-driven dysregulation of inhibitory synaptic function may represent an early pathogenic event, acting as a predisposing factor rather than a downstream consequence of neuronal loss.

Given the functional convergence of LRRK2 and α-synuclein, therapeutic modulation of LRRK2 may also have indirect effects on α-synuclein-associated pathogenic processes, potentially through shared regulation of membrane trafficking pathways. These findings support the concept that restoring trafficking-dependent regulation of synaptic receptors and transporters could represent an effective strategy to preserve circuit stability during early disease stages. From this perspective, targeting LRRK2 signaling may provide therapeutic benefits beyond neuroprotection by directly restoring synaptic homeostasis. Strategies aimed at stabilizing membrane localization of receptors and neurotransmitter transporters, such as astrocytic EAAT2, may represent complementary approaches to counteract the excitatory/inhibitory imbalance associated with LRRK2-related PD.

## Open Questions and Future Directions

Despite substantial progress in understanding LRRK2 signaling, several key aspects of its role in membrane trafficking and synaptic regulation remain unresolved. Addressing these questions will be essential to fully define how LRRK2 contributes to neuronal vulnerability and circuit dysfunction in PD.

One major unresolved issue concerns the variant-specific effects of pathogenic LRRK2 mutations. While the G2019S mutation has been extensively characterized and consistently associated with increased kinase activity, other disease-linked variants located in the ROC–COR domains may influence signaling through distinct mechanisms involving altered GTPase activity or protein–protein interactions (Lewis et al. 2007; Liao et al. 2014). Whether these mutations differentially affect trafficking pathways and membrane protein dynamics remains largely unexplored.

A second open question relates to cell-type specificity. LRRK2 is expressed in multiple brain cell populations, including neurons, astrocytes, microglia, and oligodendrocytes. Although neuronal mechanisms have received most attention, emerging evidence indicates that glial LRRK2 activity may significantly influence synaptic homeostasis through regulation of neurotransmitter clearance and inflammatory signaling (Booth et al. 2017). Dissecting the relative contributions of neuronal and glial trafficking pathways represents an important challenge for future studies.

Another area requiring further investigation is the temporal relationship between trafficking defects and neurodegeneration. Several experimental models suggest that synaptic alterations occur early in the disease process, preceding overt dopaminergic neuron loss (Beccano-Kelly et al. 2014; Matikainen-Ankney et al. 2016). Notably, evidence from postmortem PD brain tissue together with experimental models, indicates that LRRK2 kinase activity is increased in idiopathic PD and may be elevated at early stages of disease progression (Di Maio et al. 2018). This supports the idea that LRRK2-dependent alterations in membrane trafficking and synaptic function could represent early pathogenic events, rather than secondary consequences of dopaminergic neuron loss. However, the precise sequence of molecular events linking LRRK2-dependent trafficking alterations to progressive neuronal dysfunction remains unclear.

Moreover, LRRK2-dependent modulation of key membrane proteins such as EAAT2 and GABA_A_ receptors has been demonstrated in experimental systems uncoupled from dopaminergic signaling, indicating that these effects are not contingent upon dopaminergic neuron dysfunction. Thus, although PD is classically associated with the loss of dopaminergic neurons, LRRK2 activity alone may be sufficient to induce significant synaptic and cellular alterations under defined experimental conditions. This observation raises two non-mutually exclusive possibilities: first, that LRRK2 may act independently of the dopaminergic system, potentially contributing to early pathogenic events preceding dopaminergic disruption; and second, that LRRK2 regulates fundamental trafficking and signaling pathways whose dysregulation may be relevant across a broader spectrum of neurological disorders beyond PD.

Finally, the molecular determinants governing substrate selectivity of LRRK2 remain incompletely understood. Although Rab GTPases represent well-established substrates, the mechanisms controlling substrate recognition and spatial specificity of LRRK2 activity within different membrane compartments require further clarification (Steger et al. 2016; Pfeffer 2013). Understanding how LRRK2 selectively regulates trafficking pathways in specific cellular contexts may provide critical insight into the mechanisms underlying synaptic vulnerability in PD.

Addressing these questions will require integrative approaches combining structural biology, advanced imaging of membrane trafficking, and circuit-level analyses of synaptic function. Such efforts will be essential to clarify how dysregulation of membrane protein dynamics contributes to the early stages of LRRK2-associated neurodegeneration.

## Conclusions

Current evidence reframes LRRK2 as a central regulator of membrane proteostasis rather than solely a PD–associated kinase. Through coordinated control of vesicular trafficking, Rab phosphorylation, and surface protein dynamics, LRRK2 influences the spatial organization of receptors and transporters that determine synaptic efficacy across neuronal and glial compartments. By integrating intracellular transport with synaptic output, LRRK2 functions at a systems level, linking membrane homeostasis to circuit stability. Pathogenic variants disrupt this coordination, leading to selective alterations in cell-surface protein distribution that can reshape excitatory and inhibitory signaling. Importantly, these effects are not restricted to dopaminergic vulnerability, suggesting that LRRK2-dependent trafficking mechanisms may represent a broader principle of neuronal communication and disease susceptibility.

Considering LRRK2 through the lens of membrane organization shifts the conceptual model of LRRK2-associated pathology from a neuron loss-centered model to one focused on early dysregulation of surface proteome dynamics. This perspective highlights trafficking-dependent processes as potential targets for strategies aimed at restoring synaptic and circuit homeostasis.

## Data Availability

No datasets were generated or analysed during the current study.

## References

[CR1] Abe T, Kuwahara T, Suenaga S, Sakurai M, Takatori S, Iwatsubo T (2024) Lysosomal stress drives the release of pathogenic α-synuclein from macrophage lineage cells via the LRRK2-Rab10 pathway. iScience 27(2):108893. 10.1016/j.isci.2024.108893 PMC10835446 38313055

[CR2] Beccano-Kelly DA, Kuhlmann N, Tatarnikov I, Volta M, Munsie LN, Chou P, Cao LP, Han H, Tapia L, Farrer MJ, Milnerwood AJ (2014) Synaptic function is modulated by LRRK2 and glutamate release is increased in cortical neurons of G2019S LRRK2 knock-in mice. Front Cell Neurosci 8:301. 10.3389/fncel.2014.00301 PMC4176085 25309331

[CR3] Beilina A, Bonet-Ponce L, Kumaran R, Kordich JJ, Ishida M, Mamais A, Kaganovich A, Saez-Atienzar S, Gershlick DC, Roosen DA, Pellegrini L, Malkov V, Fell MJ, Harvey K, Bonifacino JS, Moore DJ, Cookson MR (2020) The Parkinson’s Disease Protein LRRK2 Interacts with the GARP Complex to Promote Retrograde Transport to the trans-Golgi Network. Cell Rep 31(5):107614. 10.1016/j.celrep.2020.107614 PMC7315779 32375042

[CR4] Berndsen K, Lis P, Yeshaw WM, Wawro PS, Nirujogi RS, Wightman M, Macartney T, Dorward M, Knebel A, Tonelli F, Pfeffer SR, Alessi DR (2019) PPM1H phosphatase counteracts LRRK2 signaling by selectively dephosphorylating Rab proteins. eLife 8:e50416. 10.7554/eLife.50416 PMC6850886 31663853

[CR5] Bonet-Ponce L, Cookson MR (2019) The role of Rab GTPases in the pathobiology of Parkinson’ disease. Curr Opin Cell Biol 59:73–80. 10.1016/j.ceb.2019.03.009 PMC6726541 31054512

[CR6] Bonet-Ponce L, Cookson MR (2022) LRRK2 recruitment, activity, and function in organelles. FEBS J 289(22):6871–6890. 10.1111/febs.16099 PMC8744135 34196120

[CR7] Booth HDE, Hirst WD, Wade-Martins R (2017) The Role of Astrocyte Dysfunction in Parkinson’s Disease Pathogenesis. Trends Neurosci 40(6):358–370. 10.1016/j.tins.2017.04.001 PMC5462417 28527591

[CR8] Brown EE, Blauwendraat C, Trinh J, Rizig M, Nalls MA, Leveille E, Ruskey JA, Jonvik H, Tan MMX, Bandres-Ciga S, Hassin-Baer S, Brockmann K, Infante J, Tolosa E, Ezquerra M, Romdhan B, Benmahdjoub S, Arezki M, Mhiri M, Hardy C (2021) Analysis of DNM3 and VAMP4 as genetic modifiers of LRRK2 Parkinson’s disease. Neurobiol Aging 97:148e17–148e24. 10.1016/j.neurobiolaging.2020.07.002. J., … International Parkinson Disease Genomics Consortium (IPDGC) PMC7762821 32873436

[CR9] Brzozowski CF, Hijaz BA, Singh V, Gcwensa NZ, Kelly K, Boyden ES, West AB, Sarkar D, Volpicelli-Daley LA (2021) Inhibition of LRRK2 kinase activity promotes anterograde axonal transport and presynaptic targeting of α-synuclein. Acta Neuropathol Commun 9(1):180. 10.1186/s40478-021-01283-7 PMC8576889 34749824

[CR10] Bu M, Farrer MJ, Khoshbouei H (2021) Dynamic control of the dopamine transporter in neurotransmission and homeostasis. NPJ Parkinson’s disease 7(1):22. 10.1038/s41531-021-00161-2 PMC7935902 33674612

[CR11] Choi I, Kim B, Byun JW, Baik SH, Huh YH, Kim JH, Mook-Jung I, Song WK, Shin JH, Seo H, Suh YH, Jou I, Park SM, Kang HC, Joe EH (2015) LRRK2 G2019S mutation attenuates microglial motility by inhibiting focal adhesion kinase. Nat Commun 6:8255. 10.1038/ncomms9255 PMC4647842 26365310

[CR12] Chung CY, Koprich JB, Siddiqi H, Isacson O (2009) Dynamic changes in presynaptic and axonal transport proteins combined with striatal neuroinflammation precede dopaminergic neuronal loss in a rat model of AAV alpha-synucleinopathy. J neuroscience: official J Soc Neurosci 29(11):3365–3373. 10.1523/JNEUROSCI.5427-08.2009 PMC2693917 19295143

[CR13] Civiero L, Vancraenenbroeck R, Belluzzi E, Beilina A, Lobbestael E, Reyniers L, Gao F, Micetic I, De Maeyer M, Bubacco L, Baekelandt V, Cookson MR, Greggio E, Taymans JM (2012) Biochemical characterization of highly purified leucine-rich repeat kinases 1 and 2 demonstrates formation of homodimers. PLoS ONE 7(8):e43472. 10.1371/journal.pone.0043472 PMC3430690 22952686

[CR14] Cookson MR (2010) The role of leucine-rich repeat kinase 2 (LRRK2) in Parkinson’s disease. Nat Rev Neurosci 11(12):791–797. 10.1038/nrn2935 PMC4662256 21088684

[CR15] Deng J, Lewis PA, Greggio E, Sluch E, Beilina A, Cookson MR (2008) Structure of the ROC domain from the Parkinson’s disease-associated leucine-rich repeat kinase 2 reveals a dimeric GTPase. Proc Natl Acad Sci USA 105(5):1499–1504. 10.1073/pnas.0709098105 PMC2234173 18230735

[CR16] Deniston CK, Salogiannis J, Mathea S, Snead DM, Lahiri I, Matyszewski M, Donosa O, Watanabe R, Böhning J, Shiau AK, Knapp S, Villa E, Reck-Peterson SL, Leschziner AE (2020) Structure of LRRK2 in Parkinson’s disease and model for microtubule interaction. Nature 588(7837):344–349. 10.1038/s41586-020-2673-2 PMC7726071 32814344

[CR17] Di Iacovo A, Aceto G, Iovino L, De Rosa R, Cinquetti R, Nielsen K, Alberio C, D’Ascenzo T, Civiero M, Bossi L, Roseti E, C (2025a) LRRK2 G2019S disrupts GABAergic signaling and shifts excitatory/inhibitory balance in the striatum bioRxiv 2025. 10.1101/2025.10.23.684189. 10.23.684189; doi

[CR18] Di Iacovo A, D’Agostino C, Bhatt M, Romanazzi T, Giovannardi S, Cinquetti R, Roseti C, Bossi E (2025b) The kinase LRRK2 is required for the physiological function and expression of the glial glutamate transporter EAAT2 (SLC1A2). J Neurochem 169(1):e16265. 10.1111/jnc.16265 PMC11629453 39655696

[CR19] Di Maio R, Hoffman EK, Rocha EM, Keeney MT, Sanders LH, De Miranda BR, Zharikov A, Van Laar A, Stepan AF, Lanz TA, Kofler JK, Burton EA, Alessi DR, Hastings TG, Greenamyre JT (2018) LRRK2 activation in idiopathic Parkinson’s disease. Sci Transl Med 10(451):eaar5429. 10.1126/scitranslmed.aar5429 PMC6344941 30045977

[CR20] Dou D, Aiken J, Holzbaur ELF (2024) RAB3 phosphorylation by pathogenic LRRK2 impairs trafficking of synaptic vesicle precursors. J Cell Biol 223(6):e202307092. 10.1083/jcb.202307092 PMC10959120 38512027

[CR21] Eguchi T, Kuwahara T, Sakurai M, Komori T, Fujimoto T, Ito G, Yoshimura SI, Harada A, Fukuda M, Koike M, Iwatsubo T (2018) LRRK2 and its substrate Rab GTPases are sequentially targeted onto stressed lysosomes and maintain their homeostasis. Proc Natl Acad Sci USA 115(39):E9115–E9124. 10.1073/pnas.1812196115 PMC6166828 30209220

[CR23] Erb ML, Moore DJ (2020) LRRK2 and the Endolysosomal System in Parkinson’s Disease. J Parkinson’s disease 10(4):1271–1291. 10.3233/JPD-202138 PMC7677880 33044192

[CR24] Filippini F, Nola S, Zahraoui A, Roger K, Esmaili M, Sun J, Wojnacki J, Vlieghe A, Bun P, Blanchon S, Rain JC, Taymans JM, Chartier-Harlin MC, Guerrera C, Galli T (2023) Secretion of VGF relies on the interplay between LRRK2 and post-Golgi v-SNAREs. Cell Rep 42(3):112221. 10.1016/j.celrep.2023.112221 36905628

[CR25] Flanagan B, McDaid L, Wade JJ, Toman M, Wong-Lin K, Harkin J (2021) A Computational Study of Astrocytic GABA Release at the Glutamatergic Synapse: EAAT-2 and GAT-3 Coupled Dynamics. Front Cell Neurosci 15:682460. 10.3389/fncel.2021.682460 PMC8312685 34322000

[CR26] Gardet A, Benita Y, Li C, Sands BE, Ballester I, Stevens C, Korzenik JR, Rioux JD, Daly MJ, Xavier RJ, Podolsky DK (2010) LRRK2 is involved in the IFN-gamma response and host response to pathogens. J Immunol (Baltimore Md : 1950) 185(9):5577–5585. 10.4049/jimmunol.1000548 PMC3156100 20921534

[CR27] Ghatak S, Talantova M, McKercher SR, Lipton SA (2021) Novel Therapeutic Approach for Excitatory/Inhibitory Imbalance in Neurodevelopmental and Neurodegenerative Diseases. Annu Rev Pharmacol Toxicol 61:701–721. 10.1146/annurev-pharmtox-032320-015420 PMC13138087 32997602

[CR28] Greggio E, Jain S, Kingsbury A, Bandopadhyay R, Lewis P, Kaganovich A, van der Brug MP, Beilina A, Blackinton J, Thomas KJ, Ahmad R, Miller DW, Kesavapany S, Singleton A, Lees A, Harvey RJ, Harvey K, Cookson MR (2006) Kinase activity is required for the toxic effects of mutant LRRK2/dardarin. Neurobiol Dis 23(2):329–341. 10.1016/j.nbd.2006.04.001 16750377

[CR29] Gu XL, Long CX, Sun L, Xie C, Lin X, Cai H (2010) Astrocytic expression of Parkinson’s disease-related A53T alpha-synuclein causes neurodegeneration in mice. Mol Brain 3:12. 10.1186/1756-6606-3-12 PMC2873589 20409326

[CR30] Gupta S, Tielemans A, Guevara CA, Huntley GW, Benson DL (2024) Parkinson’s-linked LRRK2-G2019S derails AMPAR trafficking, mobility, and composition in striatum with cell-type and subunit specificity. Proc Natl Acad Sci USA 121(28):e2317833121. 10.1073/pnas.2317833121 PMC11252801 38968112

[CR31] Hardingham GE, Bading H (2010) Synaptic versus extrasynaptic NMDA receptor signalling: implications for neurodegenerative disorders. Nat Rev Neurosci 11(10):682–696. 10.1038/nrn2911 PMC2948541 20842175

[CR32] He KJ, Zhang JB, Liu JY, Zhao FL, Yao XY, Tang YT, Zhang JR, Cheng XY, Hu LF, Wang F, Liu CF (2023) LRRK2 G2019S promotes astrocytic inflammation induced by oligomeric α-synuclein through NF-κB pathway. iScience 26(11):108130. 10.1016/j.isci.2023.108130 PMC10590863 37876795

[CR33] Héja L, Barabás P, Nyitrai G, Kékesi KA, Lasztóczi B, Toke O, Tárkányi G, Madsen K, Schousboe A, Dobolyi A, Palkovits M, Kardos J (2009) Glutamate uptake triggers transporter-mediated GABA release from astrocytes. PLoS ONE 4(9):e7153. 10.1371/journal.pone.0007153 PMC2744931 19777062

[CR34] Héja L, Nyitrai G, Kékesi O, Dobolyi A, Szabó P, Fiáth R, Ulbert I, Pál-Szenthe B, Palkovits M, Kardos J (2012) Astrocytes convert network excitation to tonic inhibition of neurons. BMC Biol 10:26. 10.1186/1741-7007-10-26 PMC3342137 22420899

[CR35] Huang X, Wu C, Park Y, Long X, Hoang QQ, Liao J (2019) The Parkinson’s disease-associated mutation N1437H impairs conformational dynamics in the G domain of LRRK2. FASEB journal:official publication Federation Am Soc Experimental Biology 33(4):4814–4823. 10.1096/fj.201802031R PMC6988866 30592623

[CR36] Iovino L, Giusti V, Pischedda F, Giusto E, Plotegher N, Marte A, Battisti I, Di Iacovo A, Marku A, Piccoli G, Bandopadhyay R, Perego C, Bonifacino T, Bonanno G, Roseti C, Bossi E, Arrigoni G, Bubacco L, Greggio E, Hilfiker S, Civiero L (2022) Trafficking of the glutamate transporter is impaired in LRRK2-related Parkinson’s disease. Acta Neuropathol 144(1):81–106. 10.1007/s00401-022-02437-0 PMC9217889 35596783

[CR37] Jaleel M, Nichols RJ, Deak M, Campbell DG, Gillardon F, Knebel A, Alessi DR (2007) LRRK2 phosphorylates moesin at threonine-558: characterization of how Parkinson’s disease mutants affect kinase activity. Biochem J 405(2):307–317. 10.1042/BJ20070209 PMC1904520 17447891

[CR38] Kluss JH, Mamais A, Cookson MR (2019) LRRK2 links genetic and sporadic Parkinson’s disease. Biochem Soc Trans 47(2):651–661. 10.1042/BST20180462 PMC6563926 30837320

[CR39] Kuwahara T, Iwatsubo T (2020) The Emerging Functions of LRRK2 and Rab GTPases in the Endolysosomal System. Front NeuroSci 14:227. 10.3389/fnins.2020.00227 PMC7095371 32256311

[CR40] Lewerenz J, Maher P (2015) Chronic Glutamate Toxicity in Neurodegenerative Diseases-What is the Evidence? Front NeuroSci 9:469. 10.3389/fnins.2015.00469 PMC4679930 26733784

[CR41] Lewis PA, Greggio E, Beilina A, Jain S, Baker A, Cookson MR (2007) The R1441C mutation of LRRK2 disrupts GTP hydrolysis. Biochem Biophys Res Commun 357(3):668–671. 10.1016/j.bbrc.2007.04.006 PMC1939973 17442267

[CR43] Li X, Tan YC, Poulose S, Olanow CW, Huang XY, Yue Z (2007) Leucine-rich repeat kinase 2 (LRRK2)/PARK8 possesses GTPase activity that is altered in familial Parkinson’s disease R1441C/G mutants. J Neurochem 103(1):238–247. 10.1111/j.1471-4159.2007.04743.x PMC2827244 17623048

[CR42] Li JQ, Tan L, Yu JT (2014) The role of the LRRK2 gene in Parkinsonism. Mol neurodegeneration 9:47. 10.1186/1750-1326-9-47 PMC4246469 25391693

[CR44] Liao J, Wu CX, Burlak C, Zhang S, Sahm H, Wang M, Zhang ZY, Vogel KW, Federici M, Riddle SM, Nichols RJ, Liu D, Cookson MR, Stone TA, Hoang QQ (2014) Parkinson disease-associated mutation R1441H in LRRK2 prolongs the active state of its GTPase domain. Proc Natl Acad Sci USA 111(11):4055–4060. 10.1073/pnas.1323285111 PMC3964117 24591621

[CR45] Liu Z, Bryant N, Kumaran R, Beilina A, Abeliovich A, Cookson MR, West AB (2018) LRRK2 phosphorylates membrane-bound Rabs and is activated by GTP-bound Rab7L1 to promote recruitment to the trans-Golgi network. Hum Mol Genet 27(2):385–395. 10.1093/hmg/ddx410 PMC5886198 29177506

[CR46] Longo F, Mercatelli D, Novello S, Arcuri L, Brugnoli A, Vincenzi F, Russo I, Berti G, Mabrouk OS, Kennedy RT, Shimshek DR, Varani K, Bubacco L, Greggio E, Morari M (2017) Age-dependent dopamine transporter dysfunction and Serine129 phospho-α-synuclein overload in G2019S LRRK2 mice. Acta Neuropathol Commun 5(1):22. 10.1186/s40478-017-0426-8 PMC5351259 28292328

[CR47] Ma B, Xu L, Pan X, Sun L, Ding J, Xie C, Koliatsos VE, Cai H (2016) LRRK2 modulates microglial activity through regulation of chemokine (C-X3-C) receptor 1 -mediated signalling pathways. Hum Mol Genet 25(16):3515–3523. 10.1093/hmg/ddw194 PMC5179946 27378696

[CR48] MacLeod DA, Rhinn H, Kuwahara T, Zolin A, Di Paolo G, McCabe BD, Marder KS, Honig LS, Clark LN, Small SA, Abeliovich A (2013) RAB7L1 interacts with LRRK2 to modify intraneuronal protein sorting and Parkinson’s disease risk. Neuron 77(3):425–439. 10.1016/j.neuron.2012.11.033 PMC3646583 23395371

[CR49] Madureira M, Connor-Robson N, Wade-Martins R (2020) LRRK2: Autophagy and Lysosomal Activity. Front NeuroSci 14:498. 10.3389/fnins.2020.00498 PMC7262160 32523507

[CR50] Manzoni C, Lewis PA (2017) LRRK2 and Autophagy. Adv Neurobiol 14:89–105. 10.1007/978-3-319-49969-7_5 28353280

[CR51] Matikainen-Ankney BA, Kezunovic N, Mesias RE, Tian Y, Williams FM, Huntley GW, Benson DL (2016) Altered Development of Synapse Structure and Function in Striatum Caused by Parkinson’s Disease-Linked LRRK2-G2019S Mutation. J neuroscience: official J Soc Neurosci 36(27):7128–7141. 10.1523/JNEUROSCI.3314-15.2016 PMC4938860 27383589

[CR52] Matta S, Van Kolen K, da Cunha R, van den Bogaart G, Mandemakers W, Miskiewicz K, De Bock PJ, Morais VA, Vilain S, Haddad D, Delbroek L, Swerts J, Chávez-Gutiérrez L, Esposito G, Daneels G, Karran E, Holt M, Gevaert K, Moechars DW, De Strooper B, Verstreken P (2012) LRRK2 controls an EndoA phosphorylation cycle in synaptic endocytosis. Neuron 75(6):1008–1021. 10.1016/j.neuron.2012.08.022 22998870

[CR53] Miklossy J, Arai T, Guo JP, Klegeris A, Yu S, McGeer EG, McGeer PL (2006) LRRK2 expression in normal and pathologic human brain and in human cell lines. J Neuropathol Exp Neurol 65(10):953–963. 10.1097/01.jnen.0000235121.98052.54 PMC7185781 17021400

[CR54] Moehle MS, Webber PJ, Tse T, Sukar N, Standaert DG, DeSilva TM, Cowell RM, West AB (2012) LRRK2 inhibition attenuates microglial inflammatory responses. J neuroscience: official J Soc Neurosci 32(5):1602–1611. 10.1523/JNEUROSCI.5601-11.2012 PMC3532034 22302802

[CR55] Otis TS, Jahr CE (1998) Anion currents and predicted glutamate flux through a neuronal glutamate transporter. J neuroscience: official J Soc Neurosci 18(18):7099–7110. 10.1523/JNEUROSCI.18-18-07099.1998 PMC6793262 9736633

[CR56] Pang SY, Lo RCN, Ho PW, Liu HF, Chang EES, Leung CT, Malki Y, Choi ZY, Wong WY, Kung MH, Ramsden DB, Ho SL (2022) LRRK2, GBA and their interaction in the regulation of autophagy: implications on therapeutics in Parkinson’s disease. Translational neurodegeneration 11(1):5. 10.1186/s40035-022-00281-6 PMC8805403 35101134

[CR57] Parisiadou L, Yu J, Sgobio C, Xie C, Liu G, Sun L, Gu XL, Lin X, Crowley NA, Lovinger DM, Cai H (2014) LRRK2 regulates synaptogenesis and dopamine receptor activation through modulation of PKA activity. Nat Neurosci 17(3):367–376. 10.1038/nn.3636 PMC3989289 24464040

[CR58] Pfeffer SR (2013) Rab GTPase regulation of membrane identity. Curr Opin Cell Biol 25(4):414–419. 10.1016/j.ceb.2013.04.002 PMC3729790 23639309

[CR59] Plowey ED, Johnson JW, Steer E, Zhu W, Eisenberg DA, Valentino NM, Liu YJ, Chu CT (2014) Mutant LRRK2 enhances glutamatergic synapse activity and evokes excitotoxic dendrite degeneration. Biochim Biophys Acta 1842(9):1596–1603. 10.1016/j.bbadis.2014.05.016 PMC4144018 24874075

[CR60] Purlyte E, Dhekne HS, Sarhan AR, Gomez R, Lis P, Wightman M, Martinez TN, Tonelli F, Pfeffer SR, Alessi DR (2019) Rab29 activation of the Parkinson’s disease-associated LRRK2 kinase. EMBO J 38(2):e101237. 10.15252/embj.2018101237 PMC6331717 30647193

[CR61] Russo I, Bubacco L, Greggio E (2014) LRRK2 and neuroinflammation: partners in crime in Parkinson’s disease? J Neuroinflamm 11:52. 10.1186/1742-2094-11-52 PMC3994422 24655756

[CR62] Sears SM, Hewett SJ (2021) Influence of glutamate and GABA transport on brain excitatory/inhibitory balance. Experimental biology Med (Maywood N J) 246(9):1069–1083. 10.1177/1535370221989263 PMC8113735 33554649

[CR63] Shin N, Jeong H, Kwon J, Heo HY, Kwon JJ, Yun HJ, Kim CH, Han BS, Tong Y, Shen J, Hatano T, Hattori N, Kim KS, Chang S, Seol W (2008) LRRK2 regulates synaptic vesicle endocytosis. Exp Cell Res 314(10):2055–2065. 10.1016/j.yexcr.2008.02.015 18445495

[CR64] Song Y, Gong T, Saleh MG, Mikkelsen M, Wang G, Edden RAE (2021) Upper brainstem GABA levels in Parkinson’s disease, vol 34. Magma (New York, pp 689–696. 5 10.1007/s10334-021-00910-7 PMC9650558 33745095

[CR65] Soukup SF, Kuenen S, Vanhauwaert R, Manetsberger J, Hernández-Díaz S, Swerts J, Schoovaerts N, Vilain S, Gounko NV, Vints K, Geens A, De Strooper B, Verstreken P (2016) A LRRK2-Dependent EndophilinA Phosphoswitch Is Critical for Macroautophagy at Presynaptic Terminals. Neuron 92(4):829–844. 10.1016/j.neuron.2016.09.037 27720484

[CR66] Steger M, Tonelli F, Ito G, Davies P, Trost M, Vetter M, Wachter S, Lorentzen E, Duddy G, Wilson S, Baptista MA, Fiske BK, Fell MJ, Morrow JA, Reith AD, Alessi DR, Mann M (2016) Phosphoproteomics reveals that Parkinson’s disease kinase LRRK2 regulates a subset of Rab GTPases. eLife 5:e12813. 10.7554/eLife.12813 PMC4769169 26824392

[CR67] Sweet ES, Saunier-Rebori B, Yue Z, Blitzer RD (2015) The Parkinson’s Disease-Associated Mutation LRRK2-G2019S Impairs Synaptic Plasticity in Mouse Hippocampus. J neuroscience: official J Soc Neurosci 35(32):11190–11195. 10.1523/JNEUROSCI.0040-15.2015 PMC4532754 26269629

[CR68] Tolosa E, Vila M, Klein C, Rascol O (2020) LRRK2 in Parkinson disease: challenges of clinical trials. Nat reviews Neurol 16(2):97–107. 10.1038/s41582-019-0301-2 31980808

[CR69] Volta M, Beccano-Kelly DA, Paschall SA, Cataldi S, MacIsaac SE, Kuhlmann N, Kadgien CA, Tatarnikov I, Fox J, Khinda J, Mitchell E, Bergeron S, Melrose H, Farrer MJ, Milnerwood AJ (2017) Initial elevations in glutamate and dopamine neurotransmission decline with age, as does exploratory behavior, in LRRK2 G2019S knock-in mice. eLife 6:e28377. 10.7554/eLife.28377 PMC5633343 28930069

[CR70] Waschbüsch D, Michels H, Strassheim S, Ossendorf E, Kessler D, Gloeckner CJ, Barnekow A (2014) LRRK2 transport is regulated by its novel interacting partner Rab32. PLoS ONE 9(10):e111632. 10.1371/journal.pone.0111632 PMC4216093 25360523

[CR71] West AB (2017) Achieving neuroprotection with LRRK2 kinase inhibitors in Parkinson disease. Exp Neurol 298(Pt B);236–245. 10.1016/j.expneurol.2017.07.019 PMC5693612 28764903

[CR72] West AB, Moore DJ, Biskup S, Bugayenko A, Smith WW, Ross CA, Dawson VL, Dawson TM (2005) Parkinson’s disease-associated mutations in leucine-rich repeat kinase 2 augment kinase activity. Proc Natl Acad Sci USA 102(46):16842–16847. 10.1073/pnas.0507360102 PMC1283829 16269541

[CR73] Wójtowicz AM, Dvorzhak A, Semtner M, Grantyn R (2013) Reduced tonic inhibition in striatal output neurons from Huntington mice due to loss of astrocytic GABA release through GAT-3. Front Neural Circuits 7:188. 10.3389/fncir.2013.00188 PMC3840359 24324407

